# The Influence of Online Health Information Seeking Before a Consultation on Anxiety, Satisfaction, and Information Recall, Mediated by Patient Participation: Field Study

**DOI:** 10.2196/23670

**Published:** 2021-07-05

**Authors:** Melanie de Looper, Julia C M van Weert, Barbara C Schouten, Sifra Bolle, Eric H J Belgers, Eric H Eddes, Ellen M A Smets

**Affiliations:** 1 Amsterdam School of Communication Research University of Amsterdam Amsterdam Netherlands; 2 Minddistrict Amsterdam Netherlands; 3 Zuyderland Medical Center Sittard-Geleen Netherlands; 4 Deparment of Surgery Deventer Hospital Deventer Netherlands; 5 Department of Medical Psychology Amsterdam UMC Amsterdam Public Health Amsterdam Netherlands

**Keywords:** online health seeking, patients, aging, patient participation, memory, anxiety, patient reported outcomes, consultation, health communication, cancer

## Abstract

**Background:**

Today, many cancer patients engage in online health information seeking (OHIS). However, little is known about how patients differ in their OHIS levels. In addition, OHIS might influence patient participation during a consultation with a physician, which might mediate the effects on patient outcomes.

**Objective:**

The aim of this study is twofold: first, to provide insight into which personal characteristics and psychosocial factors affect patients’ OHIS levels and, second, to test the hypothesis that the effects of OHIS on patient outcomes are mediated by patient participation during the consultation.

**Methods:**

Patient participation was operationalized in terms of patients’ absolute word count; the relative contribution of the patient, compared with the health care provider; and the number of questions and assertions expressed during the consultation. The patient outcomes measured were anxiety after the consultation, satisfaction with the consultation, and information recall. Participants in this study were patients recently diagnosed with colorectal cancer recruited from 6 hospitals in the Netherlands (n=90). Data were collected using questionnaires and audio-recorded consultations of patients with health care providers before their surgery.

**Results:**

The results showed that younger patients, higher educated patients, patients with a monitoring coping style, and patients who experienced more cancer-related stress engaged more in OHIS. In turn, OHIS was related to patient participation in terms of the patient’s absolute word count but not to the relative contribution to the consultation or expressing questions and assertions. We did not find a relation between OHIS and anxiety and OHIS and recall mediated by patient participation. However, we found that patients’ absolute word count significantly mediated the positive association between OHIS and patients’ satisfaction with the consultation.

**Conclusions:**

Results indicate positive implications of OHIS for patients’ care experience and, therefore, the importance of helping patients engage in OHIS. However, the results also suggest that OHIS is only successful in increasing a single aspect of patient participation, which might explain the absence of relations with anxiety and recall. The results suggest that more beneficial effects on patient outcomes may be achieved when health care providers support patients in OHIS.

## Introduction

### Background

Today, the internet hosts a growing body of easily accessible cancer-related information [1]. In line with this, cancer patients increasingly engage in online health information seeking (OHIS) [2,3] about their illness and treatment [4]. OHIS about one’s health or medical condition can contribute to feeling informed, which has been positively associated with patient outcomes [5]. For instance, better informed patients score higher on affective outcomes, for example, they are more satisfied with their treatment [6-9] and feel less anxious [5,10,11]. Moreover, OHIS can positively influence cognitive outcomes, such as better information recall [12,13].

Although patients generally seek web-based health information [14-16], it can be argued that the extent to which they engage in OHIS is associated with individual differences based on demographics or psychosocial characteristics [17,18]. For instance, experiencing feelings of anxiety or stress regarding a medical diagnosis can result in more information needs [19] and information seeking to cope with them [20].

Previous research did not look at the whole path from individual differences to OHIS and, in turn, patient outcomes but mainly focused on either predictors of OHIS in terms of demographics and psychosocial factors [21-24] or outcomes of OHIS [5,25-27]. More specifically, research that looked into the effects of OHIS did not take into account what happens between OHIS and patient outcomes in terms of consultations with health care providers [5,25]. This is a noteworthy omission because patients often engage in OHIS in preparation for consultations [15,16,28], which can result in a better informed and more empowered patient who feels comfortable in taking on an active role in consultations with health care providers [9,27,29]. In turn, this may lead to more active patient participation during consultations [9,30], for example, by patients expressing more concerns and asking more questions [31].

Subsequently, patient participation can positively influence factors related to the quality of care, such as satisfaction with the consultation and understanding of health information provided [32]. In addition, researchers found that patient participation is related to lower anxiety [33], increased satisfaction [34-36], and improved information recall [13,37]. However, knowledge about whether and how the effects of OHIS on these outcomes are mediated by patient participation during consultation is lacking. Therefore, the aim of this study is to examine the demographic and psychosocial factors that can predict OHIS and how OHIS, in turn, influences patient outcomes via patient participation during consultations.

### Predictors of OHIS

Cancer patients vary in the extent to which they seek online health information. The Comprehensive Model of Information Seeking is one of the most widely adopted models to discuss factors that could influence health information seeking [22]. In this model, demographics and psychosocial factors are seen as important determinants of how much an individual is inclined to search for health information.

#### Demographics

In general, studies show that demographics such as age, education level, and gender correlate with OHIS [16]. However, results are ambiguous. For example, some have shown that younger individuals seek more online health information than older individuals [16,38-40], whereas others find that older adults tend to seek more information online than their younger counterparts [41] or find no correlations with OHIS at all [42]. Frailty, or “the risk for adverse outcomes due to losses in different domains of functioning” [43], is found to be related to a decline in patients’ self-management abilities, more so than chronological aging. Therefore, the level of frailty, also called biological age, might better predict a patient’s ability to engage in OHIS than chronological age. In addition, several studies have shown that females seek online health information more frequently than males [16,38,40,44], whereas other studies show no associations between OHIS and gender [41,42]. With respect to education level, there is some evidence that higher educated individuals seek more online health information than lower educated individuals [44]; however, other studies show no such associations [20,42,45]. Finally, the tendency to search for health information online can also differ according to one’s degree of health literacy or “the ability to perform basic reading and numerical tasks required to function in the health care environment” [46]. As described in a review study, some studies show limited evidence that people with low health literacy search less frequently for health information online, compared with people with high health literacy, whereas other studies show no differences in OHIS based on health literacy [47].

#### Psychosocial Factors

In addition to demographics, OHIS can also be explained by patients’ psychosocial characteristics such as their degree of stress or anxiety and strategies to cope with such feelings. Higher levels of fear and anxiety in cancer patients have both been associated with the tendency to avoid cancer-related information [28,48] and with increased information needs [49]. Seeking relevant health information online might help patients to deal with the feelings of anxiety, and some patients feel relieved or comforted by the information they find online [45,50]. However, cancer patients differ in their need for cancer-related information [48], based on how they cope with a health threat. Some patients prefer only a very limited amount of information (blunting coping style), whereas others prefer as much information as possible (monitoring coping style) [51-56]. As the results are inconsistent, more research is needed, resulting in research question (RQ) 1:

- RQ 1: Are cancer patients’ demographic characteristics (ie, age, gender, education level, frailty, and health literacy) and psychosocial characteristics (ie, anxiety, cancer-related stress, and information-seeking coping style) related to OHIS?

### Direct Relation of OHIS and Patient Participation

#### Patient Participation

OHIS may potentially better equip patients to participate in consultations with health care providers [57-59]. Actively participating in such consultations reflects patients’ ability and willingness to express their needs, concerns, preferences, and expectations [32]. According to the linguistic model of patient participation in care, patients need a certain repertoire of informational resources to actively communicate during medical consultations [32]. Patients with sufficient knowledge about a topic or terminology related to the topic will discuss health issues more easily with their providers [60]. Therefore, the knowledge a patient possesses, which might be gained because of OHIS, influences a patient’s ability to actively communicate and is an important factor in patient participation [29,32,61].

In addition, providing patients with an opportunity to gather information and seeking online health information can empower patients by giving them the feeling that they are better prepared for their consultations, thereby making them confident enough to actively participate during consultations [9,29]. A recent review showed that gathering online health information before a consultation resulted in patients feeling more self-assured and empowered during consultations [9].

In conclusion, seeking health information online can prepare patients for interactions with health care providers by increasing knowledge and feelings of empowerment and might, therefore, be a crucial predictor of patient participation. Therefore, we argue that more OHIS leads to greater patient participation during a consultation with a health care provider, resulting in hypothesis 1 (H1):

- H1: OHIS is positively related to cancer patients’ participation during a medical consultation.

### Indirect Relation of OHIS and Patient Outcomes: The Mediating Role of Patient Participation

Both OHIS and patient participation are believed to be important independent factors that influence affective and cognitive patient outcomes [6,62]. OHIS most likely influences these outcomes via patient participation because it can increase patients’ illness-related knowledge and feelings of empowerment, leading to more patient participation [32]. Active patient participation can, in turn, positively affect factors that indicate quality of care [32]. Indeed, studies have found that patient participation results in less anxiety [6,33], more satisfaction [34-36], and better information recall [13,37].

#### Anxiety

OHIS can positively influence emotional well-being in general, for example, by making the patient feel less stressed [5] and less anxious [10-12]. OHIS can also help patients gain knowledge about their illness [30], making them feel more empowered to discuss certain topics during consultations [9], which, in turn, can lower their stress and feelings of anxiety. If patients experience feelings of anxiety beforehand, or because of OHIS, actively participating during the consultation gives them a chance to discuss their issues with the health care provider, which might help decrease their anxiety.

**Figure 1:**
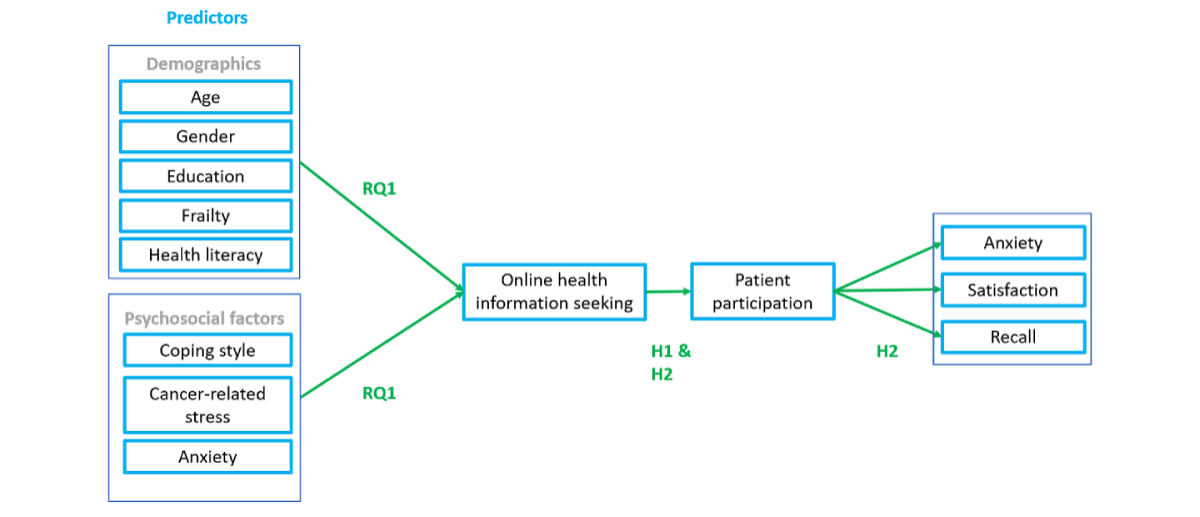
Theoretical model. H1: hypothesis 1; H2: hypothesis 2; RQ1: research question 1.

On the other hand, in some cases, OHIS can increase feelings of worry and anxiety [27,63]. Patients can experience confusion because of seeking health information [27,30], which can result in feeling less comfortable to participate and act more reserved during consultations. If a patient already feels anxious because of seeking online health information and does not actively participate during consultations, the health care provider may not be able to adequately address the patient’s anxiety. As a result, their anxiety may remain or increase even further. In line with this, we argue that the effect of OHIS on anxiety is mediated by patient participation during medical consultations (Figure 1), resulting in hypothesis 2a (H2a):

- H2a: Patient participation mediates the effect of OHIS on anxiety and stress after consultation.

#### Satisfaction

Generally, better informed patients are more satisfied with their health care processes [6,7,12,64]. Russ et al [8] found that patients who sought online health information were more satisfied with the information provided by the provider during a consultation when compared with patients who did not seek online health information.

A reason for this increase in satisfaction can be that seeking online health information before a consultation gives the patient a feeling of being prepared for the consultation [9]. Online information can help patients anticipate the discussion of certain topics during consultations or to consider possible treatments that will be proposed [65]. Knowing what to expect during the consultation can result in more active participation, including the expression of questions or expectations. These can subsequently be addressed by the health care provider, resulting in greater satisfaction with the consultation. In line with this, patients are more satisfied when providers are supportive of their search for online health information [66,67]. Therefore, it can be argued that OHIS leads to more satisfied patients through increased patient participation.

However, as discussed before, OHIS can also cause confusion, thereby inhibiting active patient participation. As a result, issues relevant to a patient may not be addressed, in which case the patient can feel disappointed and less satisfied with the consultation. Accordingly, research has shown that when the online findings do not match with the information discussed during consultation, for example, regarding diagnosis or treatment options, this can result in a less satisfied patient [68]. Therefore, we argue that the effect of OHIS on satisfaction with a consultation is mediated by patient participation (Figure 1), resulting in hypothesis 2b (H2b):

- H2b: Patient participation mediates the effect of OHIS on satisfaction with a consultation.

#### Recall

When patients engage in OHIS before a consultation and this leads to more participation during the consultation, this is likely to improve the recall of the information discussed [13,37,69-71]. One reason for the positive association between OHIS, participation, and recall is that repetition of the same information can improve information recall [72,73]. When patients search for online health information before the consultation and discuss the same information during the consultation by actively participating, this leads to a repetition in exposure to that information. In addition, exposure to a first piece of information can prime the interest for a second similar piece of information [74]. As this double exposure to the same kind of information stimulates deeper information processing, it is expected to positively influence information recall [75,76].

It can also be argued that patients who participate more actively during the consultation by asking more questions and expressing more concerns will receive more information from health care providers and are also more likely to understand the rationale and recommendations of the provider [32]. Moreover, actively participating patients are more involved and, therefore, process the information they receive during the consultations in an active manner. This active, deeper processing of information can result in better information recall [77]. Thus, we argue that the effect of OHIS on recall of the information provided during the consultation is mediated by patient participation (Figure 1), leading to hypothesis 2c (H2c):

- H2c: Patient participation mediates the effect of OHIS on information recall.

## Methods

### Design

A study was conducted in 6 Dutch hospitals among newly diagnosed colorectal cancer patients. All patients received the standard procedure of care provided by the hospitals without any alterations. All newly diagnosed patients who planned to undergo surgery were approached to participate in the study. Health care providers (surgeons and specialized nurses) and patients signed an informed consent form. Study participants received a consultation with a surgeon or specialized nurse in preparation for their surgery. This consultation was audio-recorded, transcribed, and content coded. Data were collected using questionnaires before and after the consultation.

This study was registered with Trialregister.nl (NTR5919) and was approved by the Review Board of the Amsterdam School of Communication Research (2017-PC-7979) and the medical ethical review boards of the hospitals that participated in the study (METC-nr: 13-061). The data collected to answer the RQs and hypotheses for this study were part of a larger investigation including multiple measurement moments.

### Procedure and Participants

Participants included newly diagnosed colorectal cancer patients; those who had planned to undergo surgery, possibly in combination with other treatment and had sufficient command of the Dutch language, were able to read, and had no cognitive impairment according to their medical record (eg, dementia); and those who had provided written informed consent.

Once the consultation with the surgeon was scheduled, a specialized nurse or medical secretary asked the patients if they wanted to receive study information. Patients who agreed to being contacted about this study were approached, approximately 3 days before the consultation, by the study coordinator via phone to explain what study participation would entail. Consenting patients received additional information and the first online questionnaire at time point 1 (T1) via email. Patients were asked to complete the first questionnaire 1 day before the consultation.

The scheduled consultation was recorded at time point 2a (T2a), and 2 days thereafter at time point 2b (T2b), the patients received the second questionnaire partly via email, including standard questions that were the same for all patients. Patients were also contacted via telephone 2 to 3 days after the consultation at time point 2c (T2c) by the research assistant or researcher to assess recall using recall questions that were tailored to the consultation.

**Figure 2:**
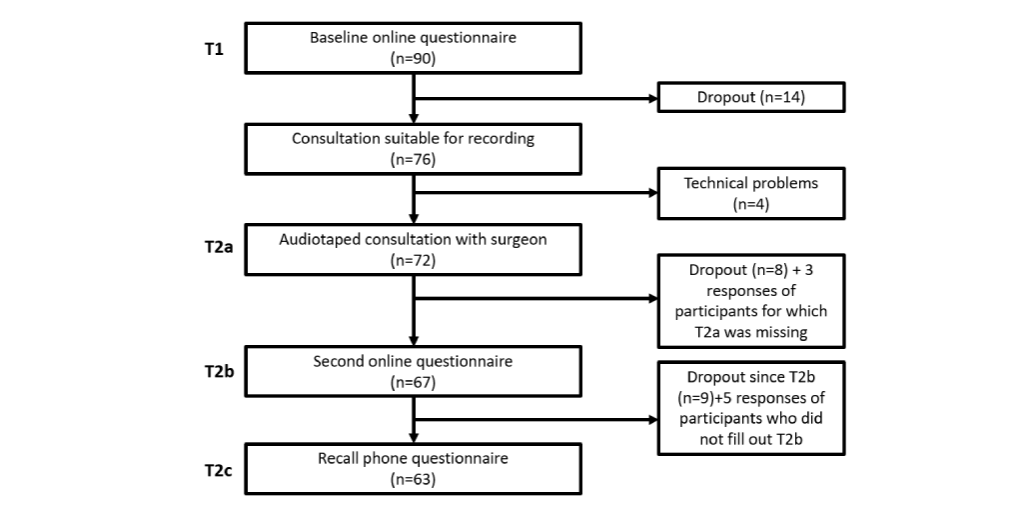
Flowchart of inclusion and dropout. T1: time point 1; T2a: time point 2a; T2b: time point 2b.

The final sample consisted of 90 patients, as seen by 23 health care providers (surgeons and specialized nurses) in 6 Dutch hospitals. During the study, 346 patients were reported to be suitable for participation by the specialized nurses or medical secretaries of the hospitals. A total of 285 patients were successfully approached to participate in the study. The other 61 patients either did not meet the inclusion criteria or could not be reached because of organizational or technical difficulties. Of the 285 patients who were successfully approached, 119 consented to participate in the study. As 29 of the consenting patients did not fill out the first questionnaire before the consultation, a total of 90 patients were included in the final analyses. Between the first and the following questionnaires, a number of patients dropped out, resulting in 72 consultation recordings, 67 responses on T2b, and 63 responses on T2c. More details about the dropout process are shown in Figure 2.

### Measures T1

#### Demographics

Sociodemographic information was obtained in the first questionnaire with questions regarding age, gender, education level, living situation, and internet use. A total of 3 categories were formed for education level (low, middle, and high). In addition, hospital records were used to obtain medical information about diagnoses and treatments of patients.

#### Frailty

Frailty was measured using the Groningen Frailty Indicator [43]. This scale contains 15 items about physical functioning (mobility, multiple health problems, fatigue, and vision or hearing problems), cognitive functioning, social functioning, and psychological functioning (feelings of depression or anxiety). The total score could range from 0 to 15; however, in this study, patients scored from 0 to 11 (mean 2.80, SD 2.45), with a higher score indicating more frailty [43].

#### Health Literacy

A 3-item questionnaire was used to measure health literacy [46]. The items addressed one’s ability to obtain and read medical information and to fill out medical forms on a 5-point scale (α=.62). The total score ranged from 1 to 5, with a higher score indicating higher health literacy (mean 4.26, SD 0.71).

#### Anxiety (Preconsultations)

Anxiety was measured at T1 using the short Dutch version of the State Trait Anxiety Inventory [10,78]. Patients rated the degree to which they were currently experiencing anxiety on a 4-point scale ranging from 1 (not at all) to 4 (very much). Higher scores on the scale indicate higher levels of anxiety. Patients scored on average 1.95 (SD 0.55), with scores ranging from 1 to 3.67. Cronbach alpha was good (α=.82).

#### Cancer-Related Stress

Cancer-related stress was measured at T1, with a subscale of the Dutch version of the Impact of Events Scale [79,80], comprising 7 items (α=.84). Participants rated the items on a 4-point Likert scale (1=*not at all*, 2=*rarely*, 3=*sometimes*, 4=*often*), with a higher score indicating higher levels of cancer-related stress. Scores ranged from 1 to 3.71, and patients scored an average of 2.03 (SD 0.70).

#### Coping Style

Coping style was measured using the adapted shortened version of the Threatening Medical Situation Inventory at T1 [81,82]. The scale consists of 3 items measuring monitoring intentions regarding the patients’ medical situation. Items addressed intentions to (1) look for information within the threatening situation, (2) go deeply into the situation by reading about it, and (3) get information from the health care provider (α=.82). Participants responded to the statements with answer options ranging from 1 (*not at all applicable to me*) to 5 (*very much applicable to me*) and scored an average of 3.46 (SD 1.07), with a higher score indicating higher monitoring intentions.

#### OHIS

On the basis of previous research [20], patients were asked to indicate on a 5-point Likert scale how often they had used the internet to seek information about their illness or treatment options before the consultation (T1). The answer options were 1 (*did not use*), 2 (*used very little*), 3 (*used sometimes*), 4 (*used regularly*), and 5 (*used often*). Patients on average scored 2.23 (SD 1.32).

### Measures T2a

#### Patient Participation

The audiotaped consultations were transcribed and manually coded by a research assistant using 3 measures to represent patient participation. This operationalization is in line with the methods used in previous research [83-86]. First, the absolute contribution of the patient to the consultation was measured using the patient’s absolute word count. Second, the relative contribution of the patient was measured by calculating the ratio of the number of words used by the patients compared with the number of words used by the health care provider. For these 2 measures, the coding process involved counting all the words used by the patient and the health care provider [83,84]. Third, the number of questions and assertions expressed by the patient during the consultation was coded using a codebook developed based on the method described by Street and Millay [32] (the complete codebook is given in Multimedia Appendix 1). A total of 10% (9/90) of the data set was double-coded by a second independent coder, resulting in acceptable intercoder reliability (κ=0.764; *P*<.001).

### Measures T2b

#### Anxiety (Postconsultation)

Anxiety was measured postconsultation (T2b) in the same manner as in the preconsultation (T1). Patients on average scored 1.80 (SD 0.66). Cronbach alpha was good (α=.86).

#### Satisfaction With the Consultation

To measure patient satisfaction with the consultation (T2b), the 5-item *Patient Satisfaction Questionnaire* was used [87]. Items addressed the following: the extent to which the patient was satisfied in terms of needs that were met by the surgeon, if the patient felt actively involved during the consultation, the information received during the consultation, the emotional support received during the consultation, and the interaction during the consultation in general (α=.80) [84]. All the answer options ranged from 1 (*not satisfied at all*) to 5 (*completely satisfied*), and patients scored an average of 4.39 (SD 0.58).

### Measures T2c

#### Information Recall

To measure information recall, the Netherlands Patient Information Recall Questionnaire (NPIRQ) [88] was used to compose the questions. The correct answers to the questions were (parts of) statements provided by the surgeon during the consultation. Therefore, the answers were literally derived from the transcribed consultations. Answers provided by the patients were scored as 0 (not recalled), 1 (partially recalled), and 2 (completely recalled). If the patient did not recall the information, there were 2 other answer option: “this information was not discussed” and “this information was discussed, but I can't remember the details,” both resulting in a score of 0 [88].

In line with the NPIRQ guidelines, a sum score was constructed by calculating the percentage of the obtained recall score (range 6%-100%) relative to the maximum achievable score (2-26 points), with higher scores indicating better recall. Patients scored an average of 60% (SD 0.19). A total of 10% of the cases (7/63) were double-coded by 2 independent coders to check intercoder reliability (mean κ=0.71; *P*<.001) [89].

### Statistical Analyses

The analyses are based on a 2-step process. First, the correlations between demographic and psychosocial variables and outcome variables were tested. The variables that significantly correlated with the outcome measures at a significance level of .10 were selected for follow-up analyses as control variables. Second, multivariate regression analyses were carried out to test whether demographic variables (age, gender, and education level) and psychosocial factors (frailty, coping style, stress, and anxiety before the consultation) were related to OHIS (RQ1) and if OHIS was related to patient participation (number of words used by the patient during the consultation, relative contribution a patient had in the consultation in terms of the word count ratio, and number of questions and assertions expressed; H1). For the mediation effects in H2a, H2b, and H2c, regression analyses using an SPSS macro allowing for mediation, (PROCESS model 4) [90] were conducted. In addition, to determine whether the relation between OHIS and the outcome variables differed depending on clustering within health care providers, multilevel analyses were carried out if the dependent variable correlated with health care providers [91].

## Results

### Sample

The age of patients included in the final analyses ranged from 39 to 88 years (mean 69.93, SD 9.93), and about two-thirds of the patients were male (59/90, 66%). Half of the patients (45/90, 50%) had a medium level of education. Patients’ health literacy was relatively high (mean 4.25, SD 0.71), and they were not frail on average (mean 2.80, SD 2.45). Almost half of the patients (41/90, 46%) indicated that they did not use the internet, 12% (11/90) used the internet very little, 21% (19/90) used the internet sometimes, 16% (14/90) used the internet regularly, and 6% (5/90) used the internet often before the consultation. Nonresponse analyses revealed that participants did not differ compared with nonparticipants regarding gender (*F*_1,309_=2.92; *P*=.09) but were on average significantly younger (mean 69.75, SD 9.93) than patients who did not wish to participate (mean 73.15, SD 10.30; *F*_1,297_=7.24; *P*=.008). The background information of the participants is presented in Table 1.

**Table 1:** Sample characteristics.

Background variables^a^			Patients
**Demographic information (n=90), mean (SD)**			
	Age (years)	69.93 (9.93)	
**Gender (n=90), n (%)**			
	Male	59 (66)	
	Female	31 (34)	
**Education level (n=88), n (%)**			
	Low	24 (27)	
	Medium	45 (51)	
	High	19 (22)	
**Health background information (n=90), mean (SD)**			
	Health literacy^b^	4.25 (0.71)	
	Frailty^c^	2.80 (2.45)	
**Psychosocial information (n=90), mean (SD)**			
	Coping style^d^	3.46 (1.07)	
**Online health information seeking behavior (n=90), n (%)**			
	Never	41 (46)	
	Very little	11 (12)	
	Sometimes	19 (21)	
	Regularly	14 (16)	
	Often	5 (6)	

^a^All cells add up to 100% owing to missing data.

^b^A higher score indicates higher levels of health literacy (maximum range 1-5; reported range 1-5).

^c^A higher score indicates higher frailty (maximum range 0-15; reported range 0-11).

^d^A higher score indicates a higher information-monitoring coping style (maximum range 1-5; reported range 1-5).

### Patient Participation

Recorded consultations (n=72) lasted between 4 minutes 26 seconds and 46 minutes 40 seconds, with an average duration of 20 minutes 19 seconds (SD 7.47 minutes). The number of words spoken during these consultations ranged from 488 to 6824 words (mean 2657, SD 1307.89). Patients spoke a minimum of 29 words and a maximum of 1347 words (mean 472.57, SD 295.46), whereas health care providers spoke at least 386 words and at the most 5124 words (mean 1998.83, SD 991.93). Patients scored a relative contribution to the consultation of 19.12% (472.57/2471.4) on average, ranging from 3.4% to 43.5% (SD 8.20); therefore, the ratio of health care providers ranged from 56.5% to 96.6%, with an average of 80.8% (SD 8.20).

A total of 69 patients asked at least one question, and 55 patients expressed at least one assertion. The number of questions ranged from 1 to 35 per consultation (mean 6.44, SD 6.36), and the number of assertions ranged from 1 to 10 per consultation (mean 2.30, SD 1.92). This resulted in a total number of questions and assertions ranging from 1 to 37 (mean 7.96, SD 7.03).

### Predictors of OHIS

#### Demographics

Correlation analyses showed that age was negatively related to OHIS (*r*=−0.29; *P*=.005), suggesting that an increase in age was associated with less OHIS. Education level and OHIS were positively correlated (*r*=0.37; *P*<.001), suggesting that higher educated patients engage more in OHIS. No significant correlations were found between OHIS and gender (*r*=0.01; *P*=.91), frailty (*r*=−0.10; *P*=.35), and health literacy (*r*=0.15; *P*=.14; Table 2).

**Table 2:** Correlation matrix.

Variable	1.	2.	3.	4.	5.	6.	7.	8.	9.	10.	11.	12.	13.	14.	15.	16.	17.
1. Age	^—a^																
2. Gender^b^	0.021	—															
3. Education level^c^	−0.057	0.038	—														
4. Health literacy	0.039	0.074	0.220^*^	—													
5. Frailty	−0.157	−0.002	−0.183	−0.295^**^	—												
6. Anxiety (preconsultation)	−0.286^**^	−0.016	−0.113	−0.041	0.461^**^	—											
7. Cancer-related stress	−0.294^**^	−0.156	−0.02	−0.045	0.203	0.554^**^	—										
8. Coping style	−0.205^*^	−0.099	0.231^*^	0.112	−0.115	0.013	0.198	—									
9. Health care provider	0.096	−0.172	−0.275^**^	−0.097	−0.162	0.119	0.109	−0.061	—								
10. consultation time	−0.043	0.079	−0.056	−0.076	−0.250^*^	0.088	0.06	0.127	0.509^**^	—							
11. Online health information seeking	−0.289^**^	0.012	0.369^**^	0.151	−0.096	0.183	0.361^**^	0.453^**^	−0.1	0.143	—						
12. Patient participation word count	−0.061	−0.229^*^	0.099	0.02	−0.131	0.142	0.082	0.336^**^	0.392^**^	0.525^**^	0.326^**^	—					
13. Patient participation relative contribution	−0.074	−0.103	0.086	0.077	0.111	0.039	−0.003	0.168	−0.062	−0.156	0.22	0.574^**^	—				
14. Patient participation questions and utterances	0.065	−0.258^*^	0.147	−0.076	−0.034	0.147	0.114	0.223	0.285^*^	0.330^**^	0.176	0.633^**^	0.295^**^	—			
15. Anxiety (postconsultation)	−0.067	−0.231^*^	−0.144	−0.124	0.435^**^	0.601^**^	0.511^**^	0.152	0.085	−0.031	0.238^*^	0.187	0.166	0.278^*^	—		
16. Satisfaction	0.134	−0.200	−0.174	−0.044	−0.151	−0.169	−0.121	−0.127	0.227	0.141	−0.191	0.086	−0.178	0.005	−0.360^**^	—	
17. Recall	−0.105	0.021	0.080	−0.126	0.073	0.150	0.161	−0.176	0.081	0.061	0.016	0.208	0.139	0.040	−0.018	0.345^**^	—

^a^Not applicable.

^b^Gender was dummy coded into 1=female and 2=male.

^c^Education was dummy coded into 1=low, 2=medium, and 3=high.

*P<.05, **P<.01, ***P<.001.

#### Psychosocial Factors

In addition, correlation analyses showed that cancer-related stress was positively correlated with OHIS (*r*=0.36; *P*<.001), implying that higher stress levels can result in more OHIS. There was a marginally significant positive correlation between anxiety before the consultation and OHIS (*r*=0.18; *P*=.08), suggesting that patients who report higher anxiety levels might engage more in OHIS. Regarding coping style, a positive correlation was found (*r*=0.45; *P*<.001), meaning patients with higher levels of monitoring coping style engaged more in OHIS (Table 2).

#### Regression Analyses

To test whether these variables predict OHIS, a regression analysis was conducted, including all possible predictors that significantly correlated with OHIS (age, education level, cancer-related stress, anxiety before the consultation, and coping style). The results showed that education level (*B*=0.54; *P*=.002), cancer-related stress (*B*=0.48; *P*=.02), and coping style (*B*=0.41; *P*=.001) were positively associated with OHIS. Thus, higher educated patients, patients experiencing more cancer-related stress, and patients with higher levels of a monitoring coping style more frequently engaged in OHIS. There was no relation between age and OHIS (*B*=−0.01; *P*=.24) and between anxiety before the consultation and OHIS (*B*=0.08; *P*=.74) based on the multivariate regression. To answer RQ1, education level, cancer-related stress, and coping style are positively related to OHIS.

#### Relation Between OHIS and Patient Participation During the Consultation (n=71)

The correlation analyses showed that gender was significantly related to the number of words used by the patient (*r*=−0.23; *P*=.005) and the number of questions and assertions expressed by the patient (*r*=−0.26; *P*=.003), suggesting that males used fewer words and expressed fewer questions and assertions than females. Coping style was also positively related to the number of words used by the patient (*r*=0.37; *P*=.004), indicating that patients with a more monitoring coping style used more words (Table 2). There were no significant correlations between the other variables and the number of words used, the relative contribution of a patient in the consultation in terms of the word count ratio, or the number of questions and assertions expressed by the patient.

Regression analyses were carried out to test the relation between OHIS and patient participation outcomes. On the basis of the correlation analyses, gender and coping style were included as control variables for the regression analyses regarding the number of words used by the patient and gender was included as the control variable for the regression regarding the number of questions and assertions expressed. No variables were included as control variables in the regression regarding relative contribution of the patient.

Results showed OHIS was positively related to the number of words used by the patient during the consultation (*B*=50.58; *P*=.02), when controlling for gender and coping style. The relation between OHIS and the relative contribution of the patient a patient had in the consultation in terms of the word count ratio was also significant (*B*=1.99; *P*=.02). OHIS was not related to the number of questions and assertions expressed (*B*=0.74; *P*=.26), when controlling for gender. In other words, patients who engaged more in OHIS used more words during the consultation and had a larger relative contribution to the conversation but did not express more questions and assertions. Regarding H1, we can conclude that OHIS is associated with some, albeit not all, indicators of patient participation during consultations.

#### Relation Between OHIS and Anxiety, Satisfaction, and Recall, Mediated by Patient Participation

The correlation analyses (n=90) showed that gender (*r*=−0.23; *P*=.005), frailty (*r*=−0.44; *P*<.001), anxiety before the consultation (*r*=−0.60; *P*<.001), and cancer-related stress (*r*=−0.51; *P*<.001) were significantly related to anxiety after the consultation. Gender was also significantly related to the number of words used by the patient (*r*=−0.23; *P*=.005) and the number of questions and assertions expressed by the patient (*r*=−0.26; *P*=.003), whereas coping style was also positively related to the number of words used by the patient (*r*=0.37; *P*=.004; Table 2). These variables were included as control variables in the regression analyses regarding anxiety after the consultation. Health care provider was only significantly related to satisfaction with the information (*r*=−0.23; *P*=.005). However, multilevel analyses showed the relation between OHIS and satisfaction was not dependent on health care provider (*F_1,4_*=−0.04; *P*=.35). There were no significant correlations between the other variables and satisfaction with the information or information recall. Therefore, no control variables were included in the regression analyses regarding satisfaction and recall.

#### Anxiety (n=64)

When controlling for gender, frailty, anxiety before the consultation, and cancer-related stress, OHIS was not related to anxiety after the consultation (*B*=0.07; *P*=.17). Regarding patient participation, the number of words used by the patient *B*=−0.01 *P*=.44), the relative contribution of the patient in terms of the word count ratio (*B*=0.01; *P*=.14), and the number of questions and assertions expressed by the patient (*B*=0.01; *P*=.66) were also not related to anxiety after the consultation. There was no significant mediation of OHIS on anxiety after the consultation via the number of words used by the patient, relative contribution of the patient to the consultation, or the number of questions and assertions (Table 3); thus, H2a must be rejected.

**Table 3:** Mediation analyses.

Relations			*B^a^*	SE	95% CI values	*t* test (*df*)	*P* value
**Direct effect of OHIS^b^**							
	On word count^c^		68.9740	27.8861	13.1535 to 124.7945	2.4734 (5,58)	.02
	On word count ratio^d^		1.9918	0.8473	0.2958 to 3.6879	2.3508 (5,58)	.02
	On questions and assertions^e^		0.7349	0.6469	−0.5601 to 2.099	1.1360 (5,58)	.26
	On anxiety		0.0666	0.0517	−0.0369 to 0.1701	1.2890 (8,55)	.20
	On satisfaction		−0.1029	0.0560	−0.2149 to 0.0091	−0.18377 (4,59)	.07
	On recall		−0.0203	0.0189	−0.0581 to 0.0175	−1.0747 (4,58)	.29
**Direct effects on anxiety**							
	Of word count		−0.0003	0.0003	−0.0009 to 0.0004	−0.7810 (8,55)	.44
	Of word count ratio		0.0141	0.0096	−0.0051 to 0.0333	1.4679 (8,55)	.15
	Of questions and assertions		0.0051	0.0117	−0.0184 to 0.0286	0.4365 (8,55)	.66
**Indirect effects of OHIS on anxiety**							
	Mediated by word count		−0.0174	0.0231	−0.0385 to 0.0549	N/A^f^	N/A
	Mediated by word count ratio		0.0280	0.0219	−0.0196 to 0.0694	N/A	N/A
	Mediated by questions and assertions		0.0038	0.0162	−0.0470 to 0.0225	N/A	N/A
**Direct effects on satisfaction**							
	Of word count		0.0008	0.0004	0.0001 to 0.0015	2.2207 (4,59)	.03
	Of word count ratio		−0.0223	0.0109	−0.0442 to 0.0005	−2.0487 (4,59)	.04
	Of questions and assertions		−0.0087	0.0139	−0.0365 to 0.0191	−0.6246 (4,59)	.53
**Indirect effects of OHIS on satisfaction**							
	Mediated by word count		0.0529	0.0283	0.0053 to 0.1158	N/A	N/A
	Mediated by word count ratio		−0.0319	0.0254	−0.0925 to 0.0068	N/A	N/A
	Mediated by questions and assertions		−0.0068	0.0162	−0.0268 to 0.0416	N/A	N/A
**Direct effects on recall**							
	Of word count		0.0002	0.0001	0.0000 to 0.0004	1.6737 (4,58)	.10
	Of word count ratio		0.0004	0.0036	−0.0068 to 0.0076	0.1033 (4,58)	.92
	Of questions and assertions		−0.0025	0.0047	−0.0119 to 0.0069	−0.5359 (4,58)	.59
**Indirect effects of OHIS on recall**							
	Mediated by word count		−0.0131	0.0091	−0.0029 to 0.0333	N/A	N/A
	Mediated by word count ratio		−.0004	0.0051	−0.0092 to 0.0127	N/A	N/A
	Mediated by questions and assertions		−0.0015	0.0043	−0.0084 to 0.0101	N/A	N/A

^a^*B*: Standardized β.

^b^OHIS: online health information seeking.

^c^Number of words used by the patient.

^d^Relative contribution of the patient in terms of words used by the patient compared with words used by the health care provider.

^e^Number of questions and assertions expressed by the patient.

^f^N/A: not applicable.

#### Satisfaction (n=64)

OHIS was marginally negatively related to satisfaction with the consultation directly (*B*=−0.10; *P*=.07), suggesting that the more a patient engaged in OHIS, the less satisfied the patient was with the consultation. The number of words used by the patient was positively related to satisfaction with the consultation (*B*=0.0008; *P*=.03), meaning the more words a patient used, the more satisfied a patient was. The relative contribution of the patient to the consultation in terms of the word count ratio was negatively related to satisfaction (*B*=−0.02; *P*=.05), suggesting that the higher the relative contribution of the patients (and therefore automatically the lower the contribution of the health care provider), the less satisfied the patient was. There was no significant relation between the number of questions and assertions expressed by the patient and satisfaction (*B*=−0.01; *P*=.54). The indirect relation between OHIS and satisfaction, based on the number of words used by the patient, was also significant (*B*=0.05; 95% CI 0.0053-0.1158). This means that patients who engaged in OHIS used more words during the consultations, which, in turn, was positively related to more satisfaction with the consultation. Therefore, H2b is partly supported.

#### Recall

The analyses showed no significant correlation between OHIS and information recall (*B*=−0.02; *P*=.28). In addition, there was no significant relation between the number of words used by the patient (*B*=0.00; *P*=.10), the relative contribution of the patient to the consultation (*B*=0.01; *P*=.92), the number of questions and assertions expressed (*r*=−0.01; *P*=.59), and information recall. In addition, there was no significant mediation of OHIS on information recall via 1 of the patient participation measures (Table 3). This implies that H2c must be rejected.

## Discussion

### Review of Findings

The aim of this study is twofold. First, this study examined which demographic and psychosocial factors could predict OHIS of newly diagnosed cancer patients. Second, we investigated how OHIS subsequently relates to patient participation during consultations and how this, in turn, affects patients’ anxiety, satisfaction, and information recall. Regarding demographic factors, the results showed that patients with higher levels of education were more inclined to engage in OHIS. With respect to psychosocial factors, higher levels of cancer-related stress are associated with more OHIS, and patients with a monitoring coping style also engage more in OHIS. In turn, OHIS was positively related to patient participation in terms of the number of words used by the patient during the consultation and the relative contribution of the patient in the consultation but not to the number of questions and assertions expressed.

The negative direct relation between OHIS and satisfaction shows that more OHIS leads to lower patient satisfaction. In addition, the number of words used by the patient was related to higher levels of satisfaction with the consultation, whereas the relative contribution of the patient in the consultation was related to lower levels of satisfaction. The results also showed a positive indirect relation between OHIS and satisfaction via the number of words used by the patient, meaning that patients who engaged more in OHIS used more words during the consultation, which, in turn, was positively related to satisfaction with the consultation. On the basis of these results, it can be concluded that OHIS can lead to both more and less satisfaction with the consultation, depending on the mediation of the number of words used by the patient.

Our results indicate that not all patients engage in OHIS. In particular, lower educated patients search less for health information online. This is in line with previous research in which education has been shown to positively influence OHIS [92]. Therefore, concerns raised almost 20 years ago by Lenhart et al [31,93] regarding the digital divide still appear to be valid. As our findings suggest that OHIS is related to patient participation and satisfaction with the consultation, it can be seen as problematic that a group of patients still does not engage in OHIS.

Our results show different relations between the different measures of patient participation and OHIS. First, our results seem to suggest that patients who engage in OHIS are inclined to use more words during the consultation, which, in turn, results in greater satisfaction with the consultation. This mediation may occur regardless of the reaction of the health care providers. However, satisfaction with the consultation might also be influenced by the interplay between the patient and health care provider. For example, patient participation can elicit a response in the health care provider, for example, discussing more information during consultations [94-96]. On the other hand, the health care provider may disregard the patient’s input, which is more in line with studies that have shown health care providers to insufficiently meet the patient’s needs [93-95]. If the relative contribution of the patient is higher, it could mean that even though the patient uses more words, the health care provider does not respond to the patient’s input. This could explain why an increase in the relative contribution of the patient to the consultation is related to a decrease in satisfaction with the consultation.

Second, the undemonstrated relation between OHIS and the expression of questions and assertions contradicts previous research, suggesting that OHIS facilitates patients to express their needs and concerns [97-99]. One reason for this could be that online health content is often incorrect, incomplete, and biased [97] and is usually experienced by patients as difficult to comprehend [97-99]. If patients engage in OHIS but find information that confuses them, this might inhibit their expression of questions or assertions. In particular, if patients do not feel empowered and confident during the consultation, they might ask fewer questions and express less assertions. It might also be possible that patients did not find the right information to support them in asking questions or expressing assertions or that OHIS fulfilled patients’ information needs and already answered questions patients had. This could have resulted in patients asking fewer questions during consultations. On the other hand, finding ambiguous information online could also lead to confusion resulting in patients asking more questions during the consultation. We swiftly examined the content of the transcripts to obtain a better understanding of the differences in relations between OHIS and the separate indicators of patient participation. The transcripts showed that patients who used more words but did not express more questions and assertions mostly engaged in small talk and discussed side issues unrelated to their ongoing situation. This implies that patients who are more active in OHIS are also more active during consultations in terms of using more words; however, the information they found online did not seem to empower them enough to express treatment-related questions or assertions.

We expected that OHIS would result in less anxiety after the consultation (H2a), via more patient participation, but our results did not support this. The fact that OHIS did not influence the expression of questions and assertions might explain why we also did not find an indirect relation between OHIS and anxiety via patient participation, as feelings of anxiety could not be partly dismantled by discussing them with the health care provider.

The aforementioned line of reasoning may also explain why OHIS did not lead to better information recall, indirectly via patient participation. By not expressing questions or assertions, but just talking more about other subjects, more information was added to the consultation. The amount of information this added to the consultation could have overshadowed the most important information about the diagnosis and treatment. Previous research has shown that the amount of information discussed during a consultation can negatively influence recall of the information discussed [88].

### Strengths

This study is, to the best of our knowledge, the first to show a significant mediation of OHIS on satisfaction with the consultation via patient participation. Established models regarding the influence of OHIS on patient participation mainly focused on the ways in which patient participation can be increased by OHIS, for example, by increasing knowledge and feelings of empowerment [62], or how patient participation can influence patient outcomes [31,84,93]. Our findings help to connect and extend these models by linking these 2 processes together, considering both the influence of OHIS on patient participation and the relation between patient participation and patient outcomes.

A distinguishing feature of this study was the participants. Including newly diagnosed cancer patients is challenging because of the emotional burden the patients face. Therefore, another strength of this study is that we succeeded in collecting these data in a vulnerable population. The fact that this is a multicenter study, with participating patients being treated in 1 of 6 Dutch hospitals, made inclusion of the patients even harder. Although this is beneficial for the external validity of the study, differences occurred in the recruitment process between the hospitals and inclusion was more troubled in some hospitals than in others, resulting in varying inclusion rates between hospitals.

### Limitations and Future Research

First, patient participation was operationalized using only quantitative measures. Therefore, we could only draw conclusions based on the quantity of patient participation and not on the quality of patient participation. Future research should also qualitatively address patient participation during consultations to gain more insight into the content of patient participation. In addition, only the utterances of the patients were analyzed. The utterances of health care providers were only included in terms of relative contribution to the consultation but not in terms of content. As it seems plausible that patients’ communication is dependent on the interplay between the partakers in that consultation [31,84,92], it is advisable to analyze the behavior of all parties taking part in the consultation in future research. In addition, only behavioral measures were used in this study to measure patient participation. Adding measures of perceived participation would be a valuable addition and is, therefore, recommended for future research.

A limitation that could have influenced the relations with information recall is that in this study, the number of recall questions was based on the amount of information the patient received from the health care provider during the consultation. This means that the more information was provided, the more recall questions the patient had to answer. The amount of information is known to be negatively related to the ability to correctly recall this information [100,101], and a higher number of questions can mean a higher chance of making mistakes. The researchers of this study deliberately chose to tailor the recall questions to the consultations of each separate patient because asking a fixed set of recall questions meant asking questions about topics that were not discussed with the patient, which was seen as unethical. Researchers can decide on asking a maximum number of questions per topic in the case of long consultations.

Finally, as our results show that OHIS does not lead to expressing questions or utterances, we encourage researchers to further investigate the effects of other types of online health information, such as online tools specifically developed and offered to patients. Previous research has shown that online health information developed and offered to a specific patient population, including preparatory tools such as question prompt lists or information tailored to a patient’s situation, can be effective in increasing patient participation [99,100].

Practically, as we see a relation between some measures of patient participation and satisfaction, but not all, this study shows the importance of providing patients with the right tools to search for online health information that stimulates participation by means of expressing questions and utterances during consultations. In particular, because OHIS can also increase worry and confusion [27,30,63], health care providers are advised to guide patients with clear instructions on how to search for information online. For example, hospitals could provide patients with flyers, including information about which websites are reliable and which websites are not.

### Conclusions

This study showed that younger patients, higher educated patients, patients who experience more cancer-related stress, and patients with a monitoring coping style are more likely to engage in OHIS. OHIS is positively related to the patient’s absolute contribution during a consultation, which, in turn, results in the patient being more satisfied with the consultation. The results are an important addition to established models regarding the influence of OHIS.

## Appendix

Multimedia Appendix 1 Codebook of patient participation.

## Abbreviations

H1: Hypothesis 1
H2a: Hypothesis 2a
H2b: Hypothesis 2b
H2c: Hypothesis 2c
NPIRQ: Netherlands Patient Information Recall Questionnaire
OHIS: online health information seeking
RQ: research question
T1: Time point 1
T2a: Time point 2a
T2b: Time point 2b
T2c: Time point 2c
