# Supplementary material for: The Influence of Online Health Information Seeking Before a Consultation on Anxiety, Satisfaction, and Information Recall, Mediated by Patient Participation: Field Study
Source: J Med Internet Res. 2021 Jul 5;23(7):e23670. doi: 10.2196/23670 (PMC8290326; doi:10.2196/23670)
Supplement: Multimedia Appendix 1 [file jmir_v23i7e23670_app1.pdf]

Codebook patient participation

Enter variables (Observer)

- **Patient ID-number**
- **Healthcare provider ID-number**
- **Companion present (yes/no)**

General guidelines:

- When coding an utterance that takes a long time: add the code at the end of the sentence (you do not have to go all the way back to the beginning of the sentence).
- Questions and utterance expressed by the patient are only coded if it is aimed at the healthcare provider.
- We do not code non-verbal behavior of the patient, because it concerns an audio recording.

(Q) Informational question asked by the patient

1. General

Any questions (or questioning comments) that the patient asks the healthcare provider for the purpose of obtaining or clarifying information.

- Each time the coder asks him-/herself the question:
Is the purpose of this question to ...
(1) obtain information from the healthcare provider? or
(2) have information clarified by the healthcare provider?
If yes, than code as question asking.

- Questions that are not relevant to the health of the patient are coded, but classified under the modifier "Other". For example:
  - o Greetings: "How are you?"
  - o Travel plans or hobbies: "Have you ever been to Hawaii?"
  - o Procedure questions: "Is it okay if I sit here for a while?"

- Confirm and nod, indicating understanding of the information, is not coded as a question, such as "Huhuh", "Jaja", "Ok".

2. Initiative

Subsequently, it is coded where the initiative lies to ask the question. Does the patient ask the question on his/her own accord or does someone else encourage them to ask a question?

Question by patient initiated by healthcare provider: QPZ
Question by patient initiated by companion: QPC
Question by patient initiated by patient: QPP

- For example: **Z**: "Do you have any questions?" **P**: "Yes, I would like to know if you already have the results of the scan" (**QPZ**).
- Or as a third person says, **C**: "You still had a question about your diet?" **P**: "Yes, can I actually eat red meat?" (**QPC**).
- If the patient goes through a questionnaire after the healthcare provider has asked if the patient has any questions, then code only the first question as **QPZ**, and everything after as **QPP**.
- If the patient asks a question again because the health care provider did not understand it, code as **QPZ**.

Informational question codes (Q)

Subject	Initiative	Code
**Patient**	Patient initiated, the question is asked on their own initiative (or the patient encourages the partner to ask a question)<br><br>companion initiated, in response to companion, patient asks a question<br><br>Healthcare provider initiated, the question is asked at the invitation of the healthcare provider	QPP<br><br>QPC<br><br>QPZ

3. Content guidelines

- If the question has **a purpose other than** obtaining or clarifying information, the coder does **not score this as question, but as an assertive utterance.**
  - For example a question for a request: **P**: "Could the surgery be scheduled a little later, because I am on vacation?", is an assertive utterance (**A**).
  - Often it depends on the intonation and context of the question whether it can be coded as an informative question or an assertive utterance.
- When in doubt between informational questions and assertive utterances, code as informational question (**Q**).
- Do not code paraphrasing (in other words repeating what the doctor has said), only if it is clear that the patient is asking for clarification. Patients often repeat in different words and in a questioning tone what the healthcare provider has said. They ask for confirmation as to whether they have understood it correctly, so indirectly for clarification of the information. Assess these utterances in context. If these are only paraphrasing then do not code as question or utterance. **Note**: Repeating information sometimes appears to be just a patient's conversation style or to fit within the course of the conversation, and more summarizing than questioning. In this case, again we are not coding it.

Examples of informational questions

Informational questions

P: "What was the result of the CT?"

P: "How long do I have to stay in the hospital after the surgery?

P: "And will those metastases not continue?"

P: "So the big one is malicious?"

P: "Am I insured for that?"

P: "Is that good news or bad news?" "Is it curative or palliative?"

Questioning remarks

P: "If this is removed, will the lymph node biopsy also be taken?"

P: "So radiation is not necessary?"

P: "You mean cutting out more than just the tumor?

P: "So the only thing is, when it happens, I stay under control so ..." **Z**: "Exactly"

Questions for clarification

➔ <u>Only score if P wants clarification of information,</u>

P: "Could you repeat that again?"

P: "What did you say?"

P: "I don't understand"

(A) Assertive utterances

1. General
Statements in which the patient or a third party wants to make his or her position, opinion, idea or wishes about his or her health situation clear to the care provider.

- Each time the coder asks himself the question:
 (1) does the patient (or third party) give his opinion clearly? or
 (2) does the patient specifically want to draw attention to something?
If yes, than code as assertive utterance.

- Assertive utterances often include verbs such as: wish, want, desire, hope, think, find, etc. For example, **P**: 'I myself think that ...', 'I hope that ...', 'I believe that ...', 'I think that ...', 'I want that ...'
 o **Note**: Even if the patient does not get the chance to complete the sentence, we code it as an assertive statement.

- Assertive remarks often do not occur during anamnesis phase of a conversation. Providing explanations and additional information about the current situation and complaints in response to (a question from) the care provider is therefore not assertive. **Pay attention to this during T2a conversations.**
 o **Z**: "Have you had any other complaints?" **P**: "No, not really ... Oh yes, I was a bit tired in the months before I was diagnosed.
 o Keep the context in which patients provide additional information and for what purpose in mind while coding. In general, to code it as utterance, the patient should really want to bring something to the attention or get something done. The statement must add something to the facts presented by the patient or to the answer to a question.
 o Conservatively code patient statements about his or her health. Only if the coder thinks that the statement really reflects a belief will the comment be coded. It is therefore not about "observations about the patient's health status", but about "opinions emerging from the patient's personal beliefs about health" (according to Street).

- Information or complaints submitted on their own initiative, without prior request from the doctor, are coded as assertive utterances, as they set the agenda and start a new topic (see point 8).

- Past assertive actions are not coded. For example, it often happens that a patient tells the healthcare provider that he / she wanted a second opinion from the referring hospital. Only code as assertive if the healthcare provider is called upon immediately during the consultation.
 o **P**: "Yes, and then we asked for a second opinion, because we wanted to go to a university hospital." (no coding - because a thing of the past)
 o **Z**: "Did the second opinion come from you or from the hospital?" **P**: "No, it did come from me. I just want to make sure I get the right treatment with the highest chance of success. "(**APZ**)

- An assertive comment is about an opinion, not an emotion.
 o **P**: "I think the pain has gotten worse lately" (A), compared to **P**: "I am very sorry, the pain."

- Never code social talk as an assertive statement.

- Assessment of the context and the intonation of the utterance often partly determine whether it is coded as assertive.

2. Initiative

Subsequently, it is coded where the initiative lies to express an utterance.

- Assertive comments will often be made by the patient himself (**APP, ACC**).
- In some cases, the healthcare provider or the companion will provoke the comment,
    - o **Z**: "Do you have any questions?" P: "I would like to have an operation in the AMC." (**APZ**)

Subject	Initiative	Code
**Patient**	Patient initiated, the question is asked on their own initiative (or the patient encourages the companion to ask a question)<br><br>Companion initiated, in response to companion, patient asks a question<br><br>Care provider initiated, the comment is made at the invitation of the care provider	APP<br><br>APC<br><br>APZ

3. Content guidelines

A patient can express and utterance in several ways, such as by:

(1) Expressing an opinion. **P**: "The only thing I find annoying is the long waiting times." Or, **P**: "I prefer to use medication only when it is really necessary."
(2) Expressing a preference. **P**: "Surgery seems best to me."
(3) Making a suggestion. **P**: "I read elsewhere that ..."
(4) Disagree with the health care provider. **Z**: "Let's schedule the operation quickly." **P**: "But I don't want that at all." Or, P: "No, that doesn't seem like a good idea."
(5) Pause the caregiver in the middle of a sentence to make a period. **Z**: "Another option is to have it in the Flevo .." **P**: "Uuh sorry, but that doesn't seem like the best option to me, could that also be possible in the AMC?"
(6) Make a request to the healthcare provider. **P**: "Could the operation be scheduled a little later? I am on vacation in May. "
(7) Make a decision. **P**: "We're going for the surgery with a stoma. I don't want to take any chances. "
(8) Starting a new topic, unrelated to the current discussion, with which the patient wants to make a point or really want to highlight something. **P**: "I have also had a lot of pain in my stomach lately." Or, **P**: "Something else, do you already know what the result of the scan is?"

- When this happens, the patient will speak for a while. However, only the first opening line is encoded as an utterance.

(9) Expressing personal or religious views. **P**: "I pray every day, and I believe it will be all right."

More examples of assertive utterances

P: "Sorry to interrupt but ... actually I really want to know if there are any possibilities. That's what we come for, of course. Let's be honest.'
P: "Yes, exactly yes, but she was talking about an alternative method. But that will be discussed this afternoon, I think. "
P: "If I qualify, I really want to." [Join a trial]
P: "But… what I actually like to know first, do you know what the result of the scan is?"
P: "I think that [with this treatment] I have a little more chance of getting through this properly."
P: "I hope for the holidays" (on the planned treatment)
P: "Can I record the conversation?"
P: "Can I bring this [drawing / folder]?

P: "I can ask a lot of questions, but I prefer to go from phase to phase" (about dealing with the disease / information provision - under modifier "psychosocial")
